# Effects of composite probiotics on growth performance, diarrhea rate, and serum indicators in newborn Holstein calves

**DOI:** 10.5713/ab.250717

**Published:** 2026-02-06

**Authors:** Kun Yang, Na Qian, Yuanyuan Xing, Min Gao, Dabiao Li

**Affiliations:** 1 College of Animal Science, Inner Mongolia Agricultural University, Hohhot, China; 2 Institute of Animal Nutrition and Feed, Inner Mongolia Academy of Agricultural & Animal Husbandry Sciences, Hohhot, China; 3 National Center of Technology Innovation for Dairy, Hohhot, China

**Keywords:** Antioxidant Capacity, Calves, Compound Probiotic, Diarrhea Rates, Growth Performance

## Abstract

**Objective:**

The present study aimed to determine the effects of compound probiotics on growth performance, diarrhea incidence, fecal pathogenic microorganisms, and serum parameters in newborn calves.

**Methods:**

A total of 48 healthy newborn calves with similar body weights (BWs; mean±standard deviation: 37.0±2.0 kg) were randomly assigned to three groups (n = 16 per group). Control group (CON), yeast group (CP1), *Bacillus licheniformis*+yeast group (CP2). The feeding trial lasted for 60 days. Growth performance, diarrhea incidence, fecal scores, and serum parameters were assessed on days 1, 30, and 60 of the trial, while fecal samples obtained on days 15 and 30 were analyzed for pathogenic microorganism positivity.

**Results:**

Compared with CON group, calves in the CP2 group had higher BW (day 30; p<0.05), average daily gain (days 1–30; p<0.05), and feed efficiency (days 1–30; p<0.05). Compared with CON group, the incidence of diarrhea and fecal scores (days 1–60) were lower in both CP1 and CP2 groups (p<0.05). Fecal samples from these groups also exhibited reduced positive rates of pathogenic microorganisms (rotavirus, coronavirus, cryptosporidium). Notably, compared with CON group, serum antioxidant and immune markers, total antioxidant capacity, superoxide dismutase, and immunoglobulin A levels were elevated (p<0.05), whereas malondialdehyde and tumor necrosis factor-alpha levels were decreased (p<0.05), and serum diamine oxidase levels were also reduced in the CP1 and CP2 groups (p<0.05). Furthermore, compared with the CON group, calves in the CP2 group exhibited lower serum cortisol and higher growth hormone (GH) levels (p<0.05). No interactions between treatment and period were observed.

**Conclusion:**

The combination of *Bacillus licheniformis* and yeast exerts promotes newborn calves’ growth and development, reduces diarrhea incidence, enhances antioxidant and immune functions, and improves overall health.

## INTRODUCTION

The neonatal period represents the most critical physiological stage in a calf’s life. Owing to their immature digestive and immune functions, calves are particularly vulnerable to diseases such as diarrhea and infections during this stage [1]. Among these, diarrhea remains one of the most serious health challenges, as it increases calf mortality, and causes substantial economic losses to the cattle industry [2]. Consequently, effective prevention and treatment of calf diarrhea, together with strategies to enhance immune function, are of critical importance for improving animal health and promoting sustainable livestock production.

In recent years, compound probiotics have attracted growing attention from researchers worldwide. As novel, eco-friendly feed additives, they are widely applied in animal husbandry due to their safety, sustainability, and lack of residues [3,4]. These products typically contain more microbial strains, most commonly *Bacillus*, *Enterococcus*, *Lactobacillus*, *Staphylococcus*, *Streptococcus*, and *Saccharomyces cerevisiae* [5]. Compound probiotics help maintain intestinal microbial balance, while their metabolic products, including amino acids, peptides, vitamins, and nucleotides, supplement dietary nutrients. These effects collectively enhance growth performance, improve immune function, and alleviate calf diarrhea by modulating gut microbiota [6,7]. Multiple studies have shown that supplementing pre-weaned calf diets with brewer’s yeast can alleviate diarrhea, improve growth performance and feed intake, and support healthy development [8–10]. Magalhães et al [11] reported that combining *Bacillus licheniformis* and *Bacillus subtilis* enhanced calf growth performance, reduced the prevalence of pathogenic microorganisms in feces, and improved overall health. Similarly, Wang et al [12] demonstrated that diets containing *Bacillus licheniformis*, *Lactobacillus plantarum*, *Pediococcus acidilactici*, and *Pediococcus pentosaceus* strengthened immune function, promoted gastrointestinal development, and reduced diarrhea severity. However, evidence remains limited regarding whether the combined use of *Bacillus licheniformis* and yeast can alleviate diarrhea, enhance antioxidant and immune functions, and promote growth and development in neonatal calves.

Therefore, this study aimed to evaluate the effects of compound probiotics on growth performance, diarrhea incidence, nutrient digestibility, and serum immune and antioxidant indices in neonatal Holstein calves. It was hypothesized that the combined use of *Bacillus licheniformis* and yeast would enhance growth performance in newborn calves, reduce diarrhea incidence and fecal scores, decrease the prevalence of pathogenic microorganisms in feces, improve intestinal health, and strengthen both antioxidant and immune functions.

## MATERIALS AND METHODS

### Animals and experimetal design

The 60-day feeding trial was conducted from August to October 2024 at a commercial ranch in Hangjinhou Banner, Bayannur City, Inner Mongolia Autonomous Region, China (40°26′–41°13′ N, 106°34′–107°24′ E; average annual temperature: 12°C). All calves were born on this ranch and received humane care throughout the study.

Within 2 h after birth, calves were weighed, orally administered 4 L of colostrum collected from freshly calved Holstein cows, and housed individually in pens (4.5×1.5 m). A total of 48 healthy newborn Holstein bull calves (average body weight: 37.0±2.0 kg) were randomly assigned to three treatment groups (n = 16 per group) in a completely randomized design. Control group (CON): fed milk without probiotics. Yeast group (CP1): supplemented with 10 g/d of a yeast product (5 g per feeding, twice daily; the probiotic was thoroughly mixed and dissolved in milk before feeding)*. Bacillus licheniformis*+ yeast group (CP2): supplemented with 10 g/d of a *Bacillus licheniformis* and yeast blend (mixed at a 1:1 ratio; 5 g per feeding, twice daily; thoroughly dissolved in milk before feeding). Yeast and *Bacillus licheniformis* products were obtained from Xi’an Xinhanbao Biotechnology. The yeast product contained *Saccharomyces cerevisiae* at 10^7^ cfu/g with a mannan content of 1.5%, while the *Bacillus licheniformis* product contained *Bacillus licheniformis* at 10^7^ cfu/g. The supplementation levels of *Saccharomyces cerevisiae* and *Bacillus licheniformis* used in this study were determined based on the dosage ranges reported in previous studies [13–15].

The feeding trial lasted 60 days, during which calves were fed milk twice daily at 07:00 and 17:00. From days 2 to 7, each calf received 4 L of pasteurized milk per day; from days 8 to 14, 6 L per day; and from days 15 to 60, 8 L per day. Clean drinking water was available *ad libitum*. To promote early rumen development, calves were introduced to a pelleted starter feed from day 15, which was offered *ad libitum*. Feed intake was adjusted daily to ensure refusals did not exceed 10% of the offered amount. Colostrum was collected from postpartum Holstein dairy cows immediately after calving, and each calf was orally administered 4 L within 2 h of birth. Calf pens were disinfected weekly. The disinfectant was evenly sprayed over the inner and outer surfaces of the calf hutches to ensure complete coverage. The disinfectant was sourced from Lanxess following standard management practices, and efforts were made to maintain dry and hygienic conditions throughout the trial. Feeding and management procedures were kept consistent across all groups. The pelleted starter feed was provided by Inner Mongolia Muquan Yuanxing Feed, and its composition and nutrient levels are presented in Table 1.

### Measurement of calf growth performance

Calves were weighed before the morning feeding on days 1, 30, and 60 to calculate average daily gain (ADG). Average daily feed intake (ADFI) was determined by accurately recording the daily amounts of starter feed offered and refusals throughout the trial. Feed efficiency (FE) was calculated using the formula: FE = ADG/ADFI.

### Diarrhea rate, and fecal score

Calf health status was monitored daily, and fecal consistency was scored using a four-point scale as described by Magalhães et al [16], cylindrical or granular feces = 1; soft feces forming a paste-like consistency = 2; unformed feces with no separation of feces and water = 3; and liquid feces with separation of feces and water = 4. Fecal scores ≥3 were considered indicative of diarrhea, and the diarrhea rate (%) was calculated as ([total number of animals with diarrhea × number of days with diarrhea] / [total number of animals × number of days of the experiment]) × 100. Fecal observation and scoring were performed by experienced full-time staff at the ranch.

### Determination of pathogenic microorganisms

Fecal samples were collected on days 15 and 30 of the experiment to assess the presence of enteric pathogens. Ten calves were randomly selected from each group, and samples were aseptically obtained directly from the rectum of each calf prior to the morning feeding, using sterile disposable gloves to minimize contamination. Approximately 30 g of feces were collected per calf, immediately transferred into sterile cryogenic tubes, labeled, and placed on ice for temporary storage during transport to the laboratory. Samples were then stored at −20°C until further analysis. The presence of rotavirus, coronavirus, *Escherichia coli* (*E. coli*), and cryptosporidium was determined using a commercial quadruple enzyme-linked immunosorbent assay (ELISA) kit (Beijing Bio Tree Biotechnology), following the manufacturer’s instructions. Optical density was measured using a microplate reader (Thermo Fisher Scientific), and positive/negative results were interpreted according to the kit’s criteria.

### Apparent nutrient digestibility

Feed samples were collected biweekly and stored at −20°C for subsequent analysis. At the end of the trial, all samples were pooled, dried at 65°C, ground through a 40-mesh sieve, and analyzed for nutrient composition. Six calves were randomly selected from each group, and approximately 50 g of fecal samples were collected daily on days 44, 45, and 46 using rectal sampling. Fecal samples collected over the three days were mixed in equal proportions to form a composite sample. The composite sample was then divided into two portions: one portion was treated with 50 mL of 10% tartaric acid per 100 g of feces for nitrogen fixation, and the other portion was left untreated and stored at −20°C for subsequent analysis of apparent nutrient digestibility. The nitrogen-fixed samples were dried at 65°C and ground through a 40-mesh sieve. The contents of dry matter (DM), ether extract (EE), and crude protein (CP) in feed and fecal samples were determined according to the standard procedures of the AOAC International [17]. Neutral detergent fiber (NDF) and acid detergent fiber (ADF) contents were determined using an Ankom 220 Fiber Analyzer (2000i; ANKOM Technology) following the method described by Van Soest [18]. Using acid-insoluble ash (AIA) as an endogenous indicator, Analytical methods of AIA followed the Chinese national standards: GB/T 23742-2009 [19], the apparent digestibility of nutrients in the whole digestive tract was estimated according to the following principle: apparent digestibility (%) = (1−[A1×F2]/[A2×F1])×100, where A1 denotes the AIA content in the diet (%), A2 denotes the AIA content in the fecal sample (%), F1 is the nutrient content of the diet (%), and F2 is the nutrient content of the fecal sample (%).

### Blood sampling and sample analysis

At 1, 30 and 60 d of age, blood was sampled via the jugular vein into 20-mL evacuated serum tubes. For 1-day-old calves, sampling was conducted 2 h after colostrum feeding, whereas for older calves, samples were obtained during the fasting period before morning feeding. After collection, the blood was left to stand at room temperature and centrifuged at 3,500×g for 15 min to separate the serum, which was then transferred into centrifuge tubes and stored in liquid nitrogen until analysis.

Serum antioxidant indices were determined using commercial kits (Nanjing Jiancheng Bioengineering Institute) following the manufacturer’s instructions. Total antioxidant capacity (T-AOC) was measured by the colorimetric method at 520 nm, malondialdehyde (MDA) by the thiobarbituric acid (TBA) method at 532 nm, superoxide dismutase (SOD) by the hydroxylamine method at 550 nm, glutathione peroxidase (GSH-Px) by the colorimetric method at 412 nm, and catalase (CAT) by the ammonium molybdate method at 405 nm. Absorbance was determined using a UV–Vis spectrophotometer (UV-1800; Shimadzu).

Serum immune indices, including interleukin-1β (IL-1β), interleukin-2 (IL-2), interleukin-4 (IL-4), tumor necrosis factor-α (TNF-α), immunoglobulin A (IgA), immunoglobulin M (IgM), and immunoglobulin G (IgG), were measured using double-antibody sandwich ELISA kits, and following the manufacturer’s protocol (Bioassay Technology Laboratory, Shanghai, China). Intestinal barrier function markers included diamine oxidase (DAO, colorimetric method) and endotoxin (ET, chromogenic limulus amebocyte lysate method). Hormone indicators included insulin (INS, double-antibody sandwich ELISA), cortisol (Cor, competitive ELISA), and growth hormone (GH, double-antibody sandwich ELISA). These kits were purchased from Shanghai Preferred Biotechnology, and assays were conducted according to the manufacturer’s instructions. Absorbance was measured at 450 nm using a microplate reader (SpectraMax iD3; Molecular Devices).

### Statistical analysis

A completely randomized design was used for this study. Data were then analyzed using a repeated measures model with the PROC MIXED and GENMOD procedure of SAS 9.4 (SAS Institute), respectively. Following the model: Yijk1 = μ+Ai+ Bj+δ(ij)k+ABij+eijkl, where Yijk1 = dependent variable, μ = mean, Ai = fixed effect of treatment i, Bj = fixed effect of time j, δ(ij) k = random effect of calf j within treatment i, ABij = fixed effect of treatment by time interaction, eijkl = residual error. Differences among least squares means were evaluated using the PDIFF option of LSMEANS with SEM, and Tukey’s test was used for pairwise comparisons. Statistical significance was declared at p<0.05.

## RESULTS

### Effects of compound probiotics on calf growth performance

As shown in Table 2, at 30 days of age, calves in the CP2 group had higher BWs compared with those in the CON and CP1 groups (p<0.05). In addition, the ADG (1–30 d) of the CP2 group was greater than that of the other two groups (p<0.05). ADFI did not differ among groups (p>0.05). FE was numerically higher in the CP1 and CP2 groups compared with the CON group, with the CP2 group showing greater FE during 1–30 d (p<0.05).

### Effects of compound probiotics on diarrhea rate and fecal score of calves

As shown in Table 3, compared with the CON group, calves in the CP1 and CP2 groups exhibited lower diarrhea rates during both 1–30 days and 31–60 days of age (p<0.05). Although there was no difference in diarrhea rates between the CP1 and CP2 groups (p>0.05), the CP2 group had the lowest overall diarrhea rate numerically, at 7.45% over the entire 60-day trial period. Fecal scores in the CP1 and CP2 groups were lower than those in the CON group, with the CP2 group showing lower fecal scores than the CP1 group (p<0.05). Additionally, fecal scores decreased in all groups from 30–60 days compared to 1–30 days.

### Effects of composite probiotics on pathogenic microorganisms in calf feces

**Figure 1:**
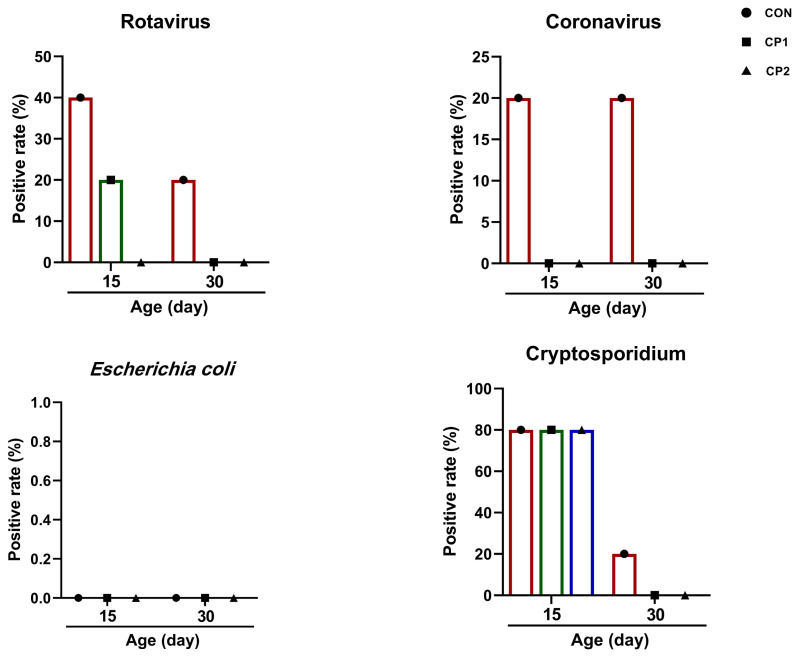
Positivity rates of rotavirus, coronavirus, *Escherichia coli*, and cryptosporidium in calf feces at 15 and 30 days of age (n = 10). CON, control group; CP1, yeast group; CP2, *Bacillus licheniformis*+yeast group. The yeast product contained *Saccharomyces cerevisiae* at 10^7^ cfu/g with a mannan content of 1.5%, while the *Bacillus licheniformis* product contained *Bacillus licheniformis* at 10^7^ cfu/g.

As shown in Figure 1, at day 15, rotavirus was detected in 40%, 20%, and 0% of calves in the CON, CP1, and CP2 groups, respectively. Coronavirus was detected only in the CON group (20%), while *E. coli* was absent in all groups. Cryptosporidium exhibited the highest prevalence, with an 80% positivity rate across all groups. By day 30, none of these pathogens were detected in the CP1 and CP2 groups.

### Effects of compound probiotics on apparent nutrient digestibility of calves

As shown in Table 4, supplementation with CP1 and CP2 increased the apparent digestibility of DM, CP, EE, and ADF compared to the CON group (p<0.05).

### Effect of compound probiotics on serum antioxidant and immune indices of calves

As shown in Table 5, compared with the CON group, serum T-AOC and SOD activities in the CP1 and CP2 groups were increased throughout the entire trial period (p<0.05), with no difference between CP1 and CP2 (p>0.05). Serum MDA levels were decreased in the CP1 group during the whole experiment compared with the CON group (p<0.05). Moreover, supplementation with CP1 and CP2 had no effects on serum IgM, IgG, IL-1β, IL-2, and IL-4 levels (p>0.05), but increased IgA levels and decreased TNF-α levels (p<0.05). There were no interactions between treatment and period.

### Effect of compound probiotics on serum diamine oxidase, endotoxin and hormone levels in calves

As shown in Table 6, throughout the experimental period, serum DAO activity was significantly lower in the CP1 and CP2 groups, with the CP2 group exhibiting the most pronounced reduction (p<0.05). No differences were observed in serum ET levels among the groups (p>0.05). Serum INS levels did not differ among the CON, CP1, and CP2 groups throughout the trial (p>0.05). However, Cor levels were lower in the CP2 group compared to the CON group (p<0.05), while growth GH levels were higher (p<0.05).

## DISCUSSION

After birth, calves exhibit underdeveloped gastrointestinal function [20]. Combined with environmental changes, pathogenic infections, and poor feeding hygiene, this immaturity reduces their resistance to external stressors. These conditions disrupt gastrointestinal microbial homeostasis, leading to diarrhea and substantial economic losses [21]. Previous studies have shown that supplementation with compound probiotics can modulate the intestinal microbiota of ruminants, increase the abundance of beneficial bacteria, improve the gut microenvironment, and enhance both antioxidant and immune functions [22–24]. The health and growth of calves before weaning directly influence their subsequent development and long-term productivity. BW, ADG, ADFI, and FE are key indicators for evaluating calf growth performance and health status. For instance, Chen et al [25] showed that dietary supplementation with 3 g/d of a probiotic complex (*Bacillus licheniformis*≥1.5×10^9^ CFU/kg; *Bacillus subtilis*≥1.0×10^9^ CFU/kg) significantly increased ADG in calves, although it had no significant effect on DMI, BW, or FE. The improvement in fecal scores increased progressively with higher dietary doses. Liu et al [26] demonstrated that dietary supplementation with composite probiotic (*Bifidobacterium animalis*, *Lactobacillus casei*, *Streptococcus faecalis*, and *Saccharomyces cerevisiae*) effectively alleviated diarrhea in suckling calves. In the present study, CP1 supplementation had no significant effect on BW, ADG, ADFI, or FE, whereas CP2 supplementation significantly increased BW (30 d), ADG (1–30 d), and FE (1–30 d). Additionally, both CP1 and CP2 supplementation significantly reduced diarrhea incidence and fecal scores compared with the CON group, with the greatest improvement observed in the CP2 group. This effect may be attributed to *Bacillus licheniformis*, which produces organic acids such as lactic acid during fermentation, thereby reducing intestinal pH, a lower pH suppresses the proliferation of pathogenic bacteria while favoring the growth of beneficial microorganisms such as *Lactobacillus* [27]. Meanwhile, the decreased intestinal pH also enhances the fermentative metabolism of *Saccharomyces cerevisiae*, resulting in increased production of mannan and glucan, these bioactive compounds further strengthen intestinal immune function and contribute to improved gut health [28]. Unfortunately, this study did not evaluate the cost implications of CP1 and CP2. However, we believe that, compared with CP1, the lower diarrhea incidence and fecal scores observed in calves receiving CP2 will translate into greater long-term savings in disease treatment costs. In future research, we will conduct a comprehensive assessment of the economic benefits associated with the composite probiotic.

To further evaluate the effects of CP1 and CP2 on pathogenic microorganisms in calf feces, we analyzed the positive rates of four common pathogens (rotavirus, coronavirus, *E. coli*, and cryptosporidium) at days 15 and 30 of the trial, as limited by cost constraints. Rotavirus, primarily transmitted via the fecal–oral route, is a major cause of acute diarrhea in calves under 60 days of age [29]. Coronaviruses, first identified in the 1960s by electron microscopy and characterized by ring-shaped surface spikes, are also frequent causes of calf diarrhea [29]. *E. coli*, a gram-negative bacterium, can induce calf diarrhea, septicemia, and peritonitis, and in severe cases may cause acute death, with incidence rates up to 90% and mortality around 50% [28]. Newborn calves, particularly those within the first week of life, are highly susceptible to pathogenic *E. coli*, leading to elevated morbidity and mortality [30]. Cryptosporidium, the most prevalent protozoan associated with infectious diarrhea, also poses a major health risk to calves [31]. Multiple studies have shown that yeast or yeast fermentation products can reduce the prevalence of *Cryptosporidium* and rotavirus in calf feces [32,33]. Similarly, Lucey et al [34] reported that the combined use of *Bacillus subtilis* and mannan oligosaccharides decreased *Cryptosporidium* prevalence in calves. Consistent with these findings, our results demonstrated that both CP1 and CP2 reduced the fecal positivity rates of coronavirus, rotavirus, and *Cryptosporidium*, with CP2 exerting the strongest effect. The underlying mechanisms may involve metabolites such as antimicrobial peptides secreted by *Bacillus licheniformis* and short-chain fatty acids generated through yeast fermentation [35,36]. These compounds can block pathogen binding to host receptors, enhance epithelial barrier function, and stimulate host immune responses, thereby suppressing the proliferation of intestinal pathogens [37,38].

A limitation of this study is the lack of a comprehensive gut microbiota analysis. Future studies integrating omics approaches are warranted to elucidate the underlying regulatory mechanisms.

In this study, we evaluated the effects of CP1 and CP2 on the apparent nutrient digestibility of newborn calves. Calves in both CP1 and CP2 groups showed significantly higher apparent digestibility of DM, CP, EE, and NDF. Wang et al [39] reported that dietary supplementation with composite probiotics (*Lactobacillus*: *Bacillus subtilis*: *Bacillus licheniformis* = 1:1:4) significantly improved the apparent digestibility of DM, CP, and EE in lambs, with NDF and ADF digestibility increasing by 17.04% and 5.71%. Similarly, Wang et al [40] found that supplementation with yeast and lactic acid bacteria enhanced the apparent digestibility of DM, NDF, and ADF in calves.

Calves in the CP1 and CP2 groups showed significantly higher serum T-AOC and SOD activity, whereas MDA levels were markedly reduced in the CP1 group. Both CP1 and CP2 supplementation significantly increased serum immunoglobulin concentrations and decreased TNF-α levels, with CP1 showing the strongest effect. No treatment×period interactions were observed. When animals are exposed to internal or external stressors, they secrete antioxidants to neutralize free radicals, thereby maintaining redox balance and protecting health [41]. T-AOC is widely used as an integrative indicator to evaluate the body’s overall antioxidant defense capacity [42]. Among enzymatic antioxidants, SOD, GSH-Px, and CAT are the major enzymes responsible for scavenging free radicals [43]. MDA, on the other hand, is a marker of lipid peroxidation and an indirect indicator of oxidative damage, where lower MDA levels correspond to stronger antioxidant capacity [44]. TNF-α, primarily produced by monocytes and macrophages, contributes to immune regulation and exhibits cytotoxic effects against tumor cells [45]. These results suggest that CP1 and CP2 enhance antioxidant capacity and immune function in calves, with CP1 being more effective. Consistent with our findings, Cai et al [46] reported that dietary supplementation with a composite probiotic (*Lactobacillus plantarum*, *Enterococcus faecium*, *Saccharomyces cerevisiae*, and *Bacillus licheniformis*) significantly increased serum antioxidant indicators in calves, including CAT (22.81%), SOD (6.49%), T-AOC (8.33%), and GSH-Px (13.67%), along with elevated IgA, IgG, and IgM levels. Similarly, Wang et al [47] showed that composite probiotic supplementation enhanced the immune function and antioxidant capacity of laying hens by elevating serum IgA and IgG, reducing MDA, and improving T-AOC. Together, these findings indicate that composite probiotics can strengthen immune responses, improve antioxidant status, and enhance disease resistance in livestock, although the underlying mechanisms warrant further study.

DAO is abundantly expressed in intestinal epithelial cells but absent in other tissues. Damage to intestinal mucosal cells results in elevated serum DAO activity. ET, a polysaccharide component of Gram-negative bacterial cell walls, can induce mucosal edema, promote villus tip necrosis, and impair intestinal barrier function [48]. To assess the protective effects of CP1 and CP2 on intestinal injury, we measured serum DAO activity and ET level in calves from different treatment groups. In contrast to previous studies [49], intestinal tissue samples were not collected to avoid additional injury. Our results showed that compared with the CON group, serum DAO activity was significantly reduced in both CP1 and CP2 supplemented calves, with the greatest reduction observed in the CP2 group, whereas serum ET levels remained unchanged. Fukuda et al [50] reported a positive correlation between serum DAO activity and diarrhea incidence in calves, and demonstrated that probiotic supplementation reduced both diarrhea and DAO activity, which is consistent with our findings. Nevertheless, the underlying mechanisms require further investigation.

Hormones are chemical messengers synthesized and secreted by endocrine cells that regulate metabolic processes in various tissues, thereby influencing growth, development, and physiological functions [51]. INS, secreted by pancreatic β-cells, lowers blood glucose by promoting glucose uptake in muscle, glycogen synthesis in the liver, lipogenesis, and overall body growth [52]. Cor is produced in response to stress, and its serum concentration is a key biomarker of stress status in calves [53]. GH regulates the growth and development of skeletal, muscular, hepatic, and adipose tissues [54]. In this study, compared with the CON group, serum insulin levels remained unchanged in calves receiving CP1 or CP2. However, calves in the CP2 group showed significantly lower Cor and higher GH levels. These findings suggest that CP2 supplementation may mitigate stress responses and promote growth and development in newborn calves. Therefore, it can be reasonably speculated that, compared with the CON and CP1 groups, calves in the CP2 group may achieve superior growth and development in later stages, potentially leading to greater savings in feeding costs.

## CONCLUSION

The results of this study demonstrate that CP1 and CP2 enhance the growth performance of newborn calves (1–30 d), alleviate diarrhea, reduce the prevalence of pathogenic microorganisms in feces, and improve apparent nutrient digestibility. In addition, both treatments enhanced serum antioxidant and immune functions, modulated hormone levels, and reduced serum DAO activity, thereby promoting growth and improving gut health.

## Figures and Tables

**Table 1:** Composition and nutrient levels of the starter (dry matter [DM] basis %)

Items	Content	Nutrient levels	Content
Corn	50.00	DM (%)	89.50
Soybean meal	32.00	CP (%DM)	23.69
Wheat bran	10.50	EE (%DM)	9.50
Barley	4.50	NDF (%DM)	8.20
Limestone	1.50	ADF (%DM)	20.20
NaCl	1.00	ADL (%DM)	36.50
Premix^1)^	1.00	Calcium (%DM)	6.95
Total	100.00	Phosphorus (%DM)	0.92

1) Premix was formulated to provide (per kilogram of the dietary DM) 52.5 mg of Zn as ZnSO_4_·7H_2_O; 30.5 mg of Mn as MnSO_4_·H_2_O; 60.5 mg of Fe as FeSO_4_·7H_2_O; 25.21 mg of Cu as CuSO_4_·5H_2_O; 0.15 mg of I as KI; 2,800 ten thousand IU of vitamin A; 4,801 IU of vitamin D; 19 ten thousand mg of vitamin E; and 3,976 mg of vitamin K_3_.

CP, crude protein; EE, ether extract; NDF, neutral detergent fiber; ADF, acid detergent fiber; ADL, acid detergent lignine.

**Table 2:** Effects of compound probiotics on BW, ADG, ADFI and FE of calves (n = 16)

Items	Treatments			SEM	p-value

	CON	CP1	CP2		
Initial BW (kg)	37.00	37.65	36.55	0.981	0.302
30 d BW (kg)	46.20^b^	47.00^b^	49.50^a^	1.010	0.041
60 d BW (kg)	76.35	75.35	75.42	1.254	0.822
ADG (1–30 d) (kg/d)	0.31^b^	0.31^b^	0.53^a^	0.021	0.023
ADG (31–60 d) (kg/d)	0.97	0.95	0.88	0.352	0.221
ADG (1–60 d) (kg/d)	0.66	0.63	0.68	0.023	0.333
ADFI (1–30 d) (kg/d)	0.17	0.14	0.14	0.061	0.152
ADFI (31–60 d) (kg/d)	0.26	0.23	0.23	0.042	0.834
ADFI (1–60 d) (kg/d)	0.22	0.20	0.19	0.020	0.504
FE (1–30 d)	1.80^b^	2.22^b^	3.80^a^	0.483	0.021
FE (31–60 d)	3.74	4.14	3.82	0.514	0.822
FE (1–60 d)	3.01	3.20	3.58	0.332	0.061

CON, control group; CP1, yeast group; CP2, *Bacillus licheniformis*+yeast group.

The yeast product contained *Saccharomyces cerevisiae* at 10^7^ cfu/g with a mannan content of 1.5%, while the *Bacillus licheniformis* product contained *Bacillus licheniformis* at 10^7^ cfu/g.

a,b Values with different lowercase letters indicate significant differences (p<0.05).

SEM, standard error of the means; BW, body weight; ADG, average daily gain; ADFI, average daily feed intake; FE, feed efficiency.

**Table 3:** Effects of compound probiotics on diarrhea and fecal score in calves (n = 16)

Items	Treatments			SEM	p-value

	CON	CP1	CP2		
Diarrhea rate (1–30 d) (%)	16.8^a^	11.0^b^	8.4^b^	3.510	0.012
Diarrhea rate (30–60 d) (%)	10. 3^a^	7.3^b^	6.5^b^	2.314	0.041
Diarrhea rate (1–60 d) (%)	13.24^a^	9.20^b^	7.45^b^	2.991	0.011
Fecal score (1–30 d)	1.36^a^	1.33^b^	1.24^c^	0.056	0.022
Fecal score (30–60 d)	1.25^a^	1.22^b^	1.15^c^	0.078	0.023
Fecal score (1–60 d)	1.29^a^	1.27^b^	1.18^c^	0.069	0.022

CON, control group; CP1, yeast group; CP2, *Bacillus licheniformis*+yeast group.

The yeast product contained *Saccharomyces cerevisiae* at 10^7^ cfu/g with a mannan content of 1.5%, while the *Bacillus licheniformis* product contained *Bacillus licheniformis* at 10^7^ cfu/g.

a,b Values with different lowercase letters indicate significant differences (p<0.05).

SEM, standard error of the means.

**Table 4:** Apparent nutrient digestibility in calves supplemented with compound probiotics (n = 6)

Items	Treatments			SEM	p-value

	CON	CP1	CP2		
DM (%)	85.61^b^	86.09^a^	86.49^a^	0.190	0.040
CP (%)	70.33^b^	71.90^a^	73.57^a^	0.881	0.041
EE (%)	77.43^b^	78.23^a^	79.13^a^	0.432	0.032
NDF (%)	65.10	66.20	66.53	0.563	0.721
ADF (%)	43.43^b^	45.10^a^	45.93^a^	0.322	0.013

CON, control group; CP1, yeast group; CP2, *Bacillus licheniformis*+yeast group.

The yeast product contained *Saccharomyces cerevisiae* at 10^7^ cfu/g with a mannan content of 1.5%, while the *Bacillus licheniformis* product contained *Bacillus licheniformis* at 10^7^ cfu/g.

a,b Values with different lowercase superscripts differ significantly (p<0.05).

SEM, standard error of the means; DM, dry matter; CP, crude protein; EE, ether extract; NDF, neutral detergent fiber; ADF, acid detergent fiber.

**Table 5:** Effects of compound probiotics on serum antioxidant and immune indices of calves (n = 16)

Items	Treatments			SEM	p-value		
	
	CON	CP1	CP2		T	P	T×P
Antioxidant indices							
T-AOC (U/mL)	12.99^b^	13.41^a^	13.52^a^	0.251	0.020	0.009	0.112
SOD (U/mL)	97.55^b^	111.94^a^	113.47^a^	3.732	0.030	0.002	0.293
GSH-Px (nmol/mL)	78.21	82.74	79.14	4.621	0.791	0.201	0.091
CAT (U/mL)	10.08	10.25	10.41	0.293	0.322	0.122	0.064
MDA (nmol/mL)	1,975.79^a^	1,886.53^b^	1,906.51^a^	53.313	0.032	0.001	0.110
Immune indices							
IgA (μg/mL)	230.25^a^	232.74^b^	234.23^b^	8.322	0.031	0.002	0.080
IgM (μg/mL)	136.56	138.86	132.62	4.990	0.252	0.001	0.101
IgG (μg/mL)	2,025.03	2,221.79	2,121.94	69.423	0.440	0.060	0.320
IL-1β (ng/L)	60.30	60.44	59.87	1.232	0.051	0.080	0.150
IL-2 (ng/L)	154.89	185.14	127.81	5.110	0.372	0.352	0.572
IL-4 (ng/L)	67.09	72.49	69.59	2.980	0.492	0.061	0.452
TNF-α (ng/L)	140.16^a^	127.50^b^	126.35^b^	6.650	0.041	0.333	0.931

CON, control group; CP1, yeast group; CP2, *Bacillus licheniformis*+yeast group.

T represents the treatment effect, P represents the period effect (days 1, 30, and 60 of the study period), and T×P represents the interaction between treatment and period.

The yeast product contained *Saccharomyces cerevisiae* at 10^7^ cfu/g with a mannan content of 1.5%, while the *Bacillus licheniformis* product contained *Bacillus licheniformis* at 10^7^ cfu/g.

a,b Values with different lowercase letters are significantly different (p<0.05).

SEM, standard error of the means; T-AOC, total antioxidant capacity; SOD, superoxide dismutase; GSH-Px, glutathione peroxidase; CAT, catalase; MDA, malondialdehyde; IgA, immunoglobulin A; IgM, immunoglobulin M; IgG, immunoglobulin G; IL-1β, interleukin-1 beta; IL-2, interleukin-2; IL-4, interleukin-4; TNF-α, tumor necrosis factor-alpha.

**Table 6:** Effects of compound probiotics on serum DAO activity, ET and hormone levels in calves (n = 16)

Items	Treatments			SEM	p-value		
	
	CON	CP1	CP2		T	P	T×P
DAO (pg/mL)	154.55^a^	145.12^b^	136.86^c^	6.210	0.021	0.014	0.061
ET (ng/L)	376.56	441.92	403.2	3.733	0.052	0.032	0.071
INS (mIU/L)	57.01	51.87	55.81	1.371	0.432	0.630	0.803
Cor (μg/L)	158.80^a^	143.62^a^	128.59^b^	6.852	0.041	0.512	0.292
GH (μg/L)	42.51^a^	43.81^a^	46.13^b^	1.010	0.031	0.600	0.334

CON, control group; CP1, yeast group; CP2, *Bacillus licheniformis*+yeast group.

T represents the treatment effect, P represents the period effect (days 1, 30, and 60 of the study period), and T×P represents the interaction between treatment and period.

The yeast product contained *Saccharomyces cerevisiae* at 10^7^ cfu/g with a mannan content of 1.5%, while the *Bacillus licheniformis* product contained *Bacillus licheniformis* at 10^7^ cfu/g.

a–c Values with different lowercase letters are significantly different (p<0.05).

DAO, diamine oxidase; ET, endotoxin; SEM, standard error of the means; INS, insulin; Cor, cortisol; GH, growth hormone.

## Data Availability

Upon reasonable request, the datasets of this study can be available from the corresponding author.

## References

[1] Zhai Y Kim M Fan P Machine learning-enhanced assessment of potential probiotics from healthy calves for the treatment of neonatal calf diarrhea Front Microbiol 2024 15 1507537 10.3389/fmicb.2024.1507537 39717273 PMC11663915

[2] Bernal-Córdoba C Branco-Lopes R Alonso-López Y Antimicrobial drugs used in the prevention and control of protozoal and bacterial calf diarrhea: a scoping review Prev Vet Med 2025 241 1065243 10.1016/j.prevetmed.2025.106543 40319541

[3] Li Z Dai X Yang F Compound probiotics promote the growth of piglets through activating the JAK2/STAT5 signaling pathway Front Microbiol 2025 16 1480077 10.3389/fmicb.2025.1480077 40432965 PMC12107631

[4] Wang Y Zhang C Chen X Dietary supplementation of compound probiotics to improve performance, egg quality, biochemical parameters and intestinal morphology of laying hens Front Vet Sci 2024 11 1505151 10.3389/fvets.2024.1505151 39776595 PMC11703898

[5] Li J Li H Zhou Y Effects of compound probiotics on cecal microbiota and metabolome of swine Animals 2023 13 1006 10.3390/ani13061006 36978547 PMC10044668

[6] Feng Y Chen Y Zuo Z Effects of compound probiotics on intestinal and liver transcriptome, immunity and disease resistance of zig-zag eel (Mastacembelus armatus) Aquaculture 2025 609 742836 10.1016/j.aquaculture.2025.742836

[7] Liu SR Li Z Wu K Effects of a probiotic complex on fecal scoring in lactating calves with diarrhea Chin Dairy Sci 2023 2 15 8

[8] Lesmeister KE Heinrichs AJ Gabler MT Effects of supplemental yeast (Saccharomyces cerevisiae) culture on rumen development, growth characteristics, and blood parameters in neonatal dairy calves J Dairy Sci 2004 87 1832 9 10.3168/jds.S0022-0302(04)73340-8 15453499

[9] Salah N Legendre H Nenov V Does micro-granulated yeast probiotic (Saccharomyces cerevisiae) supplementation in milk replacer affect health, growth, feed efficiency and economic gain of calves? Vet Anim Sci 2024 23 100329 10.1016/j.vas.2023.100329 38222799 PMC10787290

[10] Brewer MT Anderson KL Yoon I Scott MF Carlson SA Amelioration of salmonellosis in pre-weaned dairy calves fed Saccharomyces cerevisiae fermentation products in feed and milk replacer Vet Microbiol 2014 172 248 55 10.1016/j.vetmic.2014.05.026 24954478

[11] Magalhães J Cappellozza BI dos Santos TC Effects of supplementing direct-fed microbials on health and growth of preweaning Gyr × Holstein dairy calves J Dairy Sci 2024 107 6117 30 10.3168/jds.2023-24434 38608942

[12] Wang H Yu Z Gao Z Effects of compound probiotics on growth performance, rumen fermentation, blood parameters, and health status of neonatal Holstein calves J Dairy Sci 2022 105 2190 200 10.3168/jds.2021-20721 34955257

[13] Kosenda K Yabashi E Takeda S Ohtsuka H Effect of live yeast Saccharomyces cerevisiae supplementation on immune factors in Japanese Black calves during the growth periods J Vet Med Sci 2023 85 290 5 10.1292/JVMS.22-0437 36682803 PMC10076186

[14] Zheng WC Hao XY Zhang CX Effects of Saccharomyces cerevisiae and Bacillus licheniformis on in vitro rumen fermentation of sheep Chin Anim Husb Vet Med 2019 46 3208 15 10.16431/j.cnki.1671-7236.2019.11.009

[15] Ramírez JF Medina S García N Effects of the supplementation with yeast (saccharomyces cerevisiae) on weight gain and development of water buffalo calves Ital J Anim Sci 2016 6 505 7 10.4081/ijas.2007.s2.505

[16] Magalhães VJA Susca F Lima FS Branco AF Yoon I Santos JEP Effect of feeding yeast culture on performance, health, and immunocompetence of dairy calves J Dairy Sci 2008 91 1497 509 10.3168/jds.2007-0582 18349243

[17] Association of Official Analytical Chemists AOAC International Official methods of analysis 18th ed AOAC International 2005

[18] Van Soest PJ Nutritional ecology of the ruminant 2nd ed Cornell University Press 1994

[19] National Technical Committee on Feed Industry of Standardization Administration of China (SAC/TC 76) GB/T 23742-2009 [Internet]. SAC/TC 76; 2009 [cited 2025 Aug 1]. Available from: https://kns.cnki.net/kcms2/article/abstract?v=VrduTR4bJX4rfGP0q4cLeH23-UakmvBwU8JsAZu1gDbyUo64NrjkX-LbegB5ZCH68_yxVUolqm8hEVRBvHbxE_EbMgjF8i2RcHL6Fqgtl0cWOUOn6GiFlQTcBBaxQ2yKEnvQL2XG7tnalEZD-ntN30FoVTbcl0SvmYohkpWMCIlJHIGGm3Orxg==&uniplatform=NZKPT&language=CHS

[20] Nishihara K Villot C Cangiano L Guan LL Steele M Bacteria colonization and gene expression related to immune function in colon mucosa is associated with growth in neonatal calves regardless of live yeast supplementation J Anim Sci Biotechnol 2024 15 76 10.1186/S40104-024-01030-7 38835065 PMC11151515

[21] Huang Q Ma F Jin Y Gao D Chang M Sun P The dynamic distribution of the rectal microbiota in Holstein dairy calves provides a framework for understanding early-life gut health Anim Nutr 2024 19 301 12 10.1016/j.aninu.2024.06.007 39640550 PMC11617247

[22] Zhang FS Ma BY Gao ZH Supplementing exogenous xylanase improves the liver antioxidant capacity and immune response of Tibetan sheep fed wheat-based diets Ital J Anim Sci 2024 23 1524 34 10.1080/1828051X.2024.2411413

[23] Zhou G Liang X He X Compound enzyme preparation supplementation improves the production performance of goats by regulating rumen microbiota Appl Microbiol Biotechnol 2023 107 7287 99 10.1007/s00253-023-12804-w 37750915

[24] Liu S Yang L Zhang Y Review of yeast culture concerning the interactions between gut microbiota and young ruminant animals Front Vet Sci 2024 11 1335765 10.3389/fvets.2024.1335765 38496306 PMC10940410

[25] Chen MY An JJ Jia JJ Jia PJ Effects of adding a probiotic complex to the diet on growth performance, serum parameters, and rumen fermentation in Simmental calves China Feed 2024 13 76 9

[26] Liu B Wang C Simujide H Compound probiotics improve the diarrhea rate and intestinal microbiota of newborn calves Animals 2022 12 322 10.3390/ani12030322 35158646 PMC8833761

[27] Zhu L Zhang X Yang Z Combined supplementation of essential oils, Saccharomyces cerevisiae and isomaltooligosaccharides improves intestinal absorption and immune functions in weaned piglets J Anim Sci 2025 103 skaf391 10.1093/JAS/SKAF391 41208033 PMC12622370

[28] Provost C Yanes H Mosnier G Emergence of multidrug-resistant Escherichia coli harbouring the CS31A virulence factor in neonatal calf diarrhoea in central france Animals 2025 15 2844 10.3390/ANI15192844 41096439 PMC12523274

[29] Hou X Niu Z Wu S Dairy cattle infection with bovine rotavirus at different growth stages and its impact on health and productivity Animals 2025 15 1628 10.3390/ani15111628 40509094 PMC12153802

[30] Zhang D Zhang H Xu H Clinical symptoms and molecular epidemiology of diarrhea-causing Escherichia coli in calves Bull Microbiol 2025 52 736 48

[31] Veshkini A Kühn C Dengler F Cryptosporidium parvum infection alters the intestinal mucosa transcriptome in neonatal calves: impacts on epithelial barriers and transcellular transport systems Front Cell Infect Microbiol 2024 14 1495309 10.3389/fcimb.2024.1495309 39703373 PMC11656319

[32] Vélez J Lange MK Zieger P Yoon I Failing K Bauer C Long-term use of yeast fermentation products in comparison to halofuginone for the control of cryptosporidiosis in neonatal calves Vet Parasitol 2019 269 57 64 10.1016/j.vetpar.2019.04.008 31079829 PMC7117046

[33] Kayasaki F Okagawa T Konnai S Direct evidence of the preventive effect of milk replacer–based probiotic feeding in calves against severe diarrhea Vet Microbiol 2021 254 108976 10.1016/j.vetmic.2020.108976 33453627

[34] Lucey PM Lean IJ Aly SS Effects of mannan-oligosaccharide and Bacillus subtilis supplementation to preweaning Holstein dairy heifers on body weight gain, diarrhea, and shedding of fecal pathogens J Dairy Sci 2021 104 4290 302 10.3168/jds.2020-19425 33752289

[35] Sermkaew N Atipairin A Wanganuttara T A novel bacitracin-like peptide from mangrove-isolated Bacillus paralicheniformis NNS4-3 against MRSA and its genomic insights Antibiotics 2024 13 716 10.3390/ANTIBIOTICS13080716 39200016 PMC11350868

[36] Lei Q Cheng Z Jiang M Effects of saccharomyces cerevisiae fermentation products on growth performance, fecal short chain fatty acids, and microbiota of pre-weaning calves Anim Biosci 2025 38 955 67 10.5713/ab.24.0340 39483010 PMC12062800

[37] Ramirez-Olea H Reyes-Ballesteros B Chavez-Santoscoy RA Potential application of the probiotic Bacillus licheniformis as an adjuvant in the treatment of diseases in humans and animals: a systematic review Front Microbiol 2022 13 993451 10.3389/fmicb.2022.993451 36225361 PMC9549136

[38] Du W Wang X Hu M Modulating gastrointestinal microbiota to alleviate diarrhea in calves Front Microbiol 2023 14 1181545 10.3389/fmicb.2023.1181545 37362944 PMC10286795

[39] Wang H Zhao M Zuo Y Effects of a probiotic complex on apparent nutrient digestibility, rumen fermentation parameters, and microbial flora in lambs Chin J Anim Nutr 2025 37 3180 93

[40] Wang W Dang G Hao W Dietary supplementation of compound probiotics improves intestinal health by modulated microbiota and its SCFA products as alternatives to in-feed antibiotics Probiotics Antimicrob Proteins 2025 17 1969 84 10.1007/s12602-024-10314-3 38904897 PMC12405047

[41] Zhang FS Ma BY Gao ZH Supplementing exogenous xylanase improves the liver antioxidant capacity and immune response of Tibetan sheep fed wheat-based diets Ital J Anim Sci 2024 23 1524 34 10.1080/1828051X.2024.2411413

[42] Pang J Liu Y Kang L Bifidobacterium animalis promotes the growth of weaning piglets by improving intestinal development, enhancing antioxidant capacity, and modulating gut microbiota Appl Environ Microbiol 2022 88 e01296 22 10.1128/aem.01296-22 36300953 PMC9680619

[43] Galasso M Gambino S Romanelli MG Donadelli M Scupoli MT Browsing the oldest antioxidant enzyme: catalase and its multiple regulation in cancer Free Radic Biol Med 2021 172 264 72 10.1016/j.freeradbiomed.2021.06.010 34129927

[44] Singh Z Karthigesu IP Singh P Kaur R Use of malondialdehyde as a biomarker for assessing oxidative stress in different disease pathologies: a review Iran J Public Health 2014 43 7 16 PMC4537634 26284218

[45] Han JH Immuno-metabolic diseases and therapeutics: molecular mechanisms via inflammasome signaling Cell Commun Signal 2025 23 373 10.1186/s12964-025-02368-9 40830886 PMC12362934

[46] Cai XH Yi P Chen XY Intake of compound probiotics accelerates the construction of immune function and gut microbiome in Holstein calves Microbiol Spectr 2024 12 e01909 23 10.1128/spectrum.01909-23 38651859 PMC11237676

[47] Wang L Zhang Y Effects of different probiotics on growth performance, digestive metabolism, and immunological indicators in calves J Anim Ecol 2024 45 39 46

[48] Yang SM Zhang XD Ma HX Value of combining the serum D-lactate, diamine oxidase, and endotoxin levels to predict gut-derived infections in cancer patients J Nutr Oncol 2023 8 101 6 10.1097/JN9.0000000000000011

[49] Yu Z Cantet JM Nair MRR A proinflammatory immune response underlies impairment of intestinal barrier function in Holstein calves exposed to heat stress J Dairy Sci 2025 108 11697 706 10.3168/jds.2025-26930 40818668

[50] Fukuda T Otsuka M Nishi K Evaluation of probiotic therapy for calf diarrhea with serum diamine oxidase activity as an indicator Jpn J Vet Res 2019 67 305 11 10.14943/jjvr.67.4.305

[51] Pisoni L Relling AE The effects of supplementing yeast fermentation products on gut permeability, hormone concentration, and growth in newborn dairy calves Transl Anim Sci 2020 4 809 21 10.1093/tas/txaa004 PMC7001108 32705006

[52] Coen SP Keogh K Byrne CJ Effect of plane of nutrition during the first 12 weeks of life on growth, metabolic and reproductive hormone concentrations, and testicular relative mRNA abundance in preweaned Holstein Friesian bull calves J Anim Sci 2021 99 skab195 10.1093/jas/skab195 34175920 PMC8355607

[53] Littlejohn BP Price DM Banta JP Prenatal transportation stress alters temperament and serum cortisol concentrations in suckling Brahman calves J Anim Sci 2016 94 602 9 10.2527/jas.2015-9635 27065130

[54] Lee JS Kacem N Kim WS 645 Effects of Saccharomyces boulardii-based feed additive on performance, hormone level, diarrhea scoring and fecal microbial population in Holstein calves experiencing heat stress J Anim Sci 2017 95 316 10.2527/asasann.2017.645

